# Supplementary material for: An open-source framework for large-scale, flexible evaluation of biomedical text mining systems
Source: J Biomed Discov Collab. 2008 Jan 29;3:1. doi: 10.1186/1747-5333-3-1 (PMC2276192; doi:10.1186/1747-5333-3-1)
Supplement: Additional file 1 — Evaluation platform source code. The additional file contains the software evaluation framework discussed in this paper. The most current release can be downloaded from SourceForge.net [1]. See the accompanying README file for instructions on installation and use. Also included in the distribution is a collection of UIMA wrappers for some commonly used BioNLP tools and annotated corpora. [file 1747-5333-3-1-S1.zip › bionlp_uima_repository-1.0.1/docs/api/src-html/edu/uchsc/ccp/util/nlp/annotation/comparison/DocumentLevelAnnotationCreator.html]

```
001    package edu.uchsc.ccp.util.nlp.annotation.comparison;
002    
003    import java.util.ArrayList;
004    import java.util.HashSet;
005    import java.util.List;
006    import java.util.Set;
007    
008    import edu.uchsc.ccp.util.nlp.annotation.AnnotationSet;
009    import edu.uchsc.ccp.util.nlp.annotation.TextAnnotation;
010    import edu.uchsc.ccp.util.nlp.mention.ClassMention;
011    import edu.uchsc.ccp.util.nlp.mention.ComplexSlotMention;
012    import edu.uchsc.ccp.util.nlp.mention.SlotMention;
013    
014    /**
015     * This class creates document-level annotations for a given type. The user can optionally require a slot to be present in order for creation to
016     * occur. This is useful for such tasks as creating document-level protein annotations where you want to require the presence of the entrez_id_slot so
017     * that gene name normalization results can be computed. By requiring the presence of the entrez_id_slot, only proteins that have such a slot will
018     * have a document-level counterpart created. If no such slot presence is requested, then document-level annotations are generated for all annotations
019     * of the specified type.
020     * 
021     * @author williamb
022     * 
023     */
024    public class DocumentLevelAnnotationCreator {
025    
026        private final boolean DEBUG = false;
027        
028        private String mentionType;
029    
030        private Set<String> requiredSlots;
031    
032        private Set<String> documentLevelAnnotationKeys;
033        
034        
035    
036        /**
037         * This DocumentLevelAnnotationCreator will generate document-level annotations for all annotations of type mentionType.
038         * 
039         * @param mentionType
040         */
041        public DocumentLevelAnnotationCreator(String mentionType) {
042            this(mentionType, null);
043        }
044    
045        /**
046         * This DocumentLevelAnnotationCreator will generate document-level annotations for only those annotations that have non-empty slots specified by
047         * the requiredSlots Set.
048         * 
049         * @param mentionType
050         * @param requiredSlots
051         */
052        public DocumentLevelAnnotationCreator(String mentionType, Set<String> requiredSlots) {
053            this.mentionType = mentionType;
054            this.requiredSlots = requiredSlots;
055        }
056    
057        /**
058         * Given a list of TextAnnotations, return a list of corresponding document-level annotations
059         * 
060         * @param inputAnnotations
061         * @return
062         */
063        public List<TextAnnotation> createDocumentLevelAnnotations(List<TextAnnotation> inputAnnotations) {
064            List<TextAnnotation> documentLevelAnnotations = new ArrayList<TextAnnotation>();
065    
066            debug("Converting annotation list into document level annotations...");
067            /*
068             * This set will hold a key representing the document-level annotations that have already been created, thus ensuring that each document-level
069             * annotation is unique.
070             */
071            documentLevelAnnotationKeys = new HashSet<String>();
072    
073            for (TextAnnotation ta : inputAnnotations) {
074                /* check to see if this annotation is of the type of interest */
075                if (ta.getClassMention().getMentionName().toLowerCase().equals(mentionType.toLowerCase())) {
076                    debug("Found a " + mentionType + " annotation");
077                    if (requiredSlots != null) {
078                        /* check to see if this annotation has non-empty required slots */
079                        boolean hasRequiredSlots = true;
080                        for (String requiredSlot : requiredSlots) {
081                            List<SlotMention> slotMentions = ta.getClassMention().getSlotMentionsByName(requiredSlot);
082                            int numSlotMentions = 0;
083                            if (slotMentions != null) {
084                                numSlotMentions = slotMentions.size();
085                            }
086                            List<ComplexSlotMention> complexSlotMentions = ta.getClassMention().getComplexSlotMentionsByName(requiredSlot);
087                            int numComplexSlotMentions = 0;
088                            if (complexSlotMentions != null) {
089                                numComplexSlotMentions = complexSlotMentions.size();
090                            }
091                            if ((numSlotMentions + numComplexSlotMentions) < 1) {
092                                hasRequiredSlots = false;
093                                debug("Annotation does not have required slots");
094                                break;
095                            }
096                        }
097                        if (hasRequiredSlots) {
098                            debug("Annotation has required slots, so creating a document-level annotation");
099                            createDocumentLevelAnnotation(ta, documentLevelAnnotations);
100                        }
101                    } else {
102                        /* no slots are required, so create the document-level annotation */
103                        debug("No slots required, so creating a document-level annotation");
104                        createDocumentLevelAnnotation(ta, documentLevelAnnotations);
105                    }
106                } else {
107                    // do nothing since this is not the mention type of interest
108                }
109            }
110    
111            return documentLevelAnnotations;
112        }
113    
114        
115        
116        public List<TextAnnotation> createDocumentLevelAnnotations(List<TextAnnotation> inputAnnotations, Set<Integer> annotatorsToIgnore) {
117            List<TextAnnotation> annotationsToKeep = new ArrayList<TextAnnotation>();
118            
119            for (TextAnnotation ta : inputAnnotations) {
120                if (!annotatorsToIgnore.contains(ta.getAnnotationID())) {
121                    annotationsToKeep.add(ta);
122                }
123            }
124            
125            return createDocumentLevelAnnotations(annotationsToKeep);
126            
127        }
128        
129        /**
130         * Convert the input TextAnnotation into a document-level annotation, check to see if this annotation already exists, if it does not, add it to
131         * the list of documentLevelAnnotations
132         * 
133         * @param ta
134         * @param documentLevelAnnotations
135         */
136        private void createDocumentLevelAnnotation(TextAnnotation ta, List<TextAnnotation> documentLevelAnnotations) {
137            AnnotationSet annotationSet = new AnnotationSet(9999,"Document-level " + ta.getClassMention().getMentionName(),"Document-level annotation set.");
138            /* Create a new class mention for this annotation- "DocumentLevel-" is appended to the class mention name */
139            ClassMention documentLevelCM = new ClassMention("DocumentLevel-" + ta.getClassMention().getMentionName());
140            for (SlotMention sm : ta.getClassMention().getSlotMentions()) {
141                documentLevelCM.addSlotMention(sm);
142            }
143            for (ComplexSlotMention csm : ta.getClassMention().getComplexSlotMentions()) {
144                documentLevelCM.addComplexSlotMention(csm);
145            }
146            /* create the document-level annotation */
147            TextAnnotation documentLevelAnnot = new TextAnnotation(0,1,"",ta.getAnnotator(),null,-1, ta.getDocumentCollectionID(),ta.getDocumentID(), ta.getDocumentSectionID(),documentLevelCM);
148            documentLevelAnnot.setAnnotationSets(ta.getAnnotationSets());
149            documentLevelAnnot.addAnnotationSet(annotationSet);
150            
151            /* now we need to log the creation of this document-level annotation, and add it to the documentLevelAnnotations list if it is new */
152            String key = documentLevelAnnot.getDocumentLevelSingleLineRepresentation(true);
153            debug("Key = " + key);
154            
155            if (!documentLevelAnnotationKeys.contains(key)) {
156                documentLevelAnnotations.add(documentLevelAnnot);
157                documentLevelAnnotationKeys.add(key);
158            } else {
159                // do nothing, we don't need to add this document-level annotation since it already exisits
160            }
161            
162        }
163        
164        
165        private void debug(String message) {
166            if (DEBUG) {
167                System.err.println("DEBUG -- DocumentLevelAnnotationCreator: " + message);
168            }
169        }
170    
171    }
```
